# Light-Curing Volumetric Shrinkage in Dimethacrylate-Based Dental Composites by Nanoindentation and PAL Study

**DOI:** 10.1186/s11671-017-1845-y

**Published:** 2017-01-25

**Authors:** Olha Shpotyuk, Stanislaw Adamiak, Elvira Bezvushko, Jozef Cebulski, Maryana Iskiv, Oleh Shpotyuk, Valentina Balitska

**Affiliations:** 1 Danylo Halytsky Lviv National Medical University, 69, Pekarska St., Lviv, 79010 Ukraine; 2 Centre for Innovation and Transfer of Natural Sciences and Engineering Knowledge, University of Rzeszow, 35-959 Rzeszow, Poland; 3 Jan Dlugosz University in Czestochowa, 13/15, Armii Krajowej St., 42200 Czestochowa, Poland; 4 Vlokh Institute of Physical Optics, 23, Dragomanov St., Lviv, 79005 Ukraine; 5 Lviv State University of Life Safety, 35, Kleparivska St., Lviv, 79007 Ukraine

**Keywords:** Composites, Nanoindentation, Filler, Light curing, Positron annihilation, Trapping

## Abstract

Light-curing volumetric shrinkage in dimethacrylate-based dental resin composites Dipol® is examined through comprehensive kinetics research employing nanoindentation measurements and nanoscale atomic-deficient study with lifetime spectroscopy of annihilating positrons. Photopolymerization kinetics determined through nanoindentation testing is shown to be described via single-exponential relaxation function with character time constants reaching respectively 15.0 and 18.7 s for nanohardness and elastic modulus. Atomic-deficient characteristics of composites are extracted from positron lifetime spectra parameterized employing unconstrained x3-term fitting. The tested photopolymerization kinetics can be adequately reflected in time-dependent changes observed in average positron lifetime (with 17.9 s time constant) and fractional free volume of positronium traps (with 18.6 s time constant). This correlation proves that fragmentation of free-volume positronium-trapping sites accompanied by partial positronium-to-positron traps conversion determines the light-curing volumetric shrinkage in the studied composites.

## Background

Dental restorative composites (DRC) based on acrylic resins, which can be substantially improved by incorporating a wide row of nanofillers (forming a so-called microhybrid DRC), form an important class of biomaterials for advanced using in dentistry practice [1].

In general, it is known that DRC are formed by three distinct phases, functionalized at extra-low atomic and subatomic length scales, these being (i) polymerizable resin, (ii) filler, and (iii) filler-resin interface [1–3]. The *resin* (i) represents organic dimerthacrylate-based monomers (bisGMA—bisphenol-A-glycidylmethacrylate, UDMA—1,6-bis[2-(methacryloyloxy)ethoxycarbonylamino]-2,4,4-trimethylhexane, D3MA—decanediol dimethacrylate, or/and TEGDMA—triethyleneglycol dimethacrylate, etc.), which are able to convert from liquid to highly cross-linked polymer state under irradiation with halogen lamp or blue light. This process occurs as free-radical photo-polymerization, the monomer matrix being essentially affected by volumetric shrinkage (internal contraction stress due to drastic change in a density of polymerized system, which may finalize in a failure of bond between DRC and tooth structure) generated in *gelation transition* from macroradicals in dimethacrylates to microgel particles in visco-elastic substrate. The *filler* (ii) is composed of constituting multi-particle entities, which typically includes tightly-packed bigger glass particles having mean sizes up to a few micrometers (ensuring easy polishing and high optical quality of polymerized DRC) combined with wide row of smaller particles (inorganic microfillers such as pyrogenic highly dispersed silica SiO_2_, zirconia ZrO_2_, titania TiO_2_) having mean sizes from a few to hundred nanometers used to prevent sedimentation of bigger particles and ensure higher hardness and toughness of polymerized DRC (thus improving wear properties and toothbrush abrasion resistance). In inorganic part of most widely used hybrid DRC, the weight content of filler particles reaches 20–30% [3]. Rich-sized filler system forms a large room for DRC improvement, including enhancing modulus, radiopacity, altering thermal expansion behavior, and reducing polymerization shrinkage. The final DRC stabilization is governed by *filler-resin interface* (iii), which serves as linkage between polymerizable moieties and filler particle surface.

Doubtless, comprehensive, and deep understanding of DRC functioning in tight connection with detailed microstructure study is a strategic line for further progress in this important field. Today, the novel analytical approaches are highly demand to fill a niche between strict *microstructure* peculiarities of advanced DRC and their *principal biomedical functionality* in clinical dentistry. These should include deeper insight in the DRC microstructure, evolving both *atomic-specific* (nanoclustering, intra- and inter-particle chemical links, nanocomposite amorphicity/crystallinity, filler crystallography, nanoparticle distribution/morphology, etc.), and *atomic-deficient* entities (low-electron density or free-volume elements, i.e. vacancy-like clusters, interfacial voids and holes, inner pores and cracks, triple junctions between neighboring nanoparticles, grain-boundaries), which overall are highly deterministic factors improving the DRC usage in biomedical practice.

As a matter of fact, it should be noted that *degree of light-activated conversion* followed by polymerization shrinkage plays a crucial role in principal functionality of most successful DRC [4]. This parameter reveals positive correlation with nanomechanical properties of DRC defined as material resistance to tip indentation (*hardness*) or relative stiffness or rigidity (*elastic modulus*) [4, 5], thus allowing its reliable and measurable in vitro description. This relatively noninvasive technique is especially important for light-cured DRC in *nanohardness* (NHD) and *elastic modulus* (the Young’s modulus) *E* mode of nanoindentation testing [5–11], allowing elimination operator errors, and reproducible controlling of whole measuring system in elastic deformation range.

From a microstructure standpoint, one of such deterministic techniques for DRC functioning is positron annihilation lifetime (PAL) spectroscopy [12, 13], which is advanced instrumentation tool grounded on space-time continuum probing for interaction between electron and its antiparticle (positron). This method is highly informative for different substances despite their chemical formulation, especially for nanoparticle-based composite systems [14–17]. The possibility to use this method for polymer/filler DRC was confirmed in a number of recent publications [18–21]. Thus, Kleczewska [18, 19] studied void structure of some commercial (Filtek Supreme, Gradia Direct) and homemade DRC employing x4-fitting procedure with fixed shortest lifetime to decompose the raw PAL spectra. This PAL-spectra treatment did not allow unambiguous determination of the channel of positron annihilation in interphase, i.e. free-volume voids at filler-matrix interface, nevertheless, some important correlations were kept between composite macro-properties and PAL parameters. In our previous publications [20, 21], we tried to stretch the PAL spectroscopy for some acrylic-type DRC affected by external influences, the results being treated employing different fitting routes. The PAL-related phenomena in these composites were shown to be hierarchically complicated, being expressed in wide diversity of decaying paths for annihilating positrons and bound positron-electron states at different atomic and subatomic levels. Thus, the polymer/filler DRC have not been too successful in previous research, mainly because of some methodological problems in correct interpretation of the obtained PAL data.

The aim of this study is to characterize the photopolymerization light-curing kinetics in polymer/filler DRC revealed in its general macroscopic appearance such as NHD and/or elastic modulus *E* measurements and structurally-intrinsic volumetric parameters extracted from PAL spectra at the example of dimerthacrylate-based DRC Dipol®.

## Methods

The Dipol® is commercially available DRC produced by Oksomat-AN Ltd., Kyiv (Ukraine), belonging to totally filled microhybrid DRC [22]. The monomer matrix of this DRC is composed of BisGMA and TEGDMA, multifunctional monomer diluents, photo-initiator/catalyst and some amine additives. The Dipol® DRC is modified with multisized highly dispersive 2000-100-20-3 nm filler phase, the finest filler fraction being spherical amorphous silica SiO_2_ reinforcing nanoparticles. The total filler loading reaches 72% in a weight allowing close-packed inner structure, thus resulting in excellent elastic properties, superior polishing ability and strength, good gloss retention, and esthetics quality. Volumetric photopolymerization shrinkage in this DRC attains only 2.2% [22].

The DRC Dipol® samples were prepared by filling an inner volume of uniform disc-shaped plastic mold (6 mm in diameter and 2 mm in thickness). The bottom end surface of this plastic disc was covered by polyethylene slice film, which was separated from the DRC specimen along with outer ring around disc just before experiments. This non-polymerized DRC was marked as D0.

Part of the prepared DRC samples were polymerized in open atmosphere at room temperature (20 ± 1 °C) by anterior light illumination from curing dental wireless source (LED.T4, SEASKY, China) emitted light in 420–480 nm spectral range with ~900 mW/cm^2^ output power density. To normalize light-curing protocol for all DRC specimens, the end of guide tip from light source was maintained just above sample surface at 7 mm distance, so light beam fully covers the surface. Under these conditions, the photo-exposure lasting 60 s was enough to ensure deeply polymerized state of DRC Dipol® (accordingly to the manufacturer instruction [22]). For detailed kinetics study, the whole photopolymerization was incremented on shorter time intervals (5-10-20-30-40-50-60 s), the polymerized DRC (5 pellets in A3 shade of each type separated from disc-shaped plastic molds) being marked as D*n* (where *n* means respective light-exposure duration). After photopolymerization, the DRC samples were stored for 1 week in ambient conditions before nanoindentation and PAL testing. So the post-irradiation polymerization input [8] was also included in the modeled kinetics. To exclude possible influence from gelation [2], the experimental data for non-polymerized D0 samples were ignored, so that we started kinetics tracing from D5 sample.

**Fig. 1:**
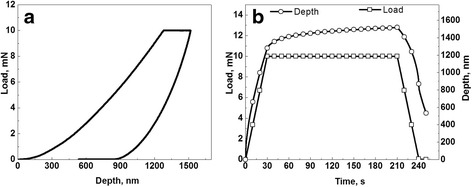
Load-displacement (**a**) and load-time (**b**) nanoindentation curves for fully-polymerized D60 probe

One nanoindentation measurement procedure exploring CSM instrument (CSM Instruments SA, Peseux, Switzerland) equipped with a Berkovitch-type (three-sided pyramidal) diamond tip was employed to study photopolymerization kinetics in the DRC Dipol®, the data collection and analysis being arranged in accordance to Oliver and Pharr method [23]. The load and displacement resolution of this CSM instrument were respectively 50 nN and 0.01 nm, the fused silica with elastic modulus of 73 GPa and Poisson’s ratio of 0.17 being used as calibration indent. The load and displacement were detected simultaneously as it reflected by load-displacement nanoindentation curve on Fig. 1a for maximal load of 10 mN and loading-unloading rate of 20 mN/min. Since the tested DRC flows under loading, especially those, which are insufficiently polymerized (Fig. 1b), the maximal loading force was applied for 180 s. Such prolongation of loading time causes some decrease in absolute NHD values, but it allows to perform stable measurements at the end of this period. Therefore, each measuring cycle includes three segments (Fig. 1a), these being loading with 20 mN/min rate, peak-load holding during 180 s, and unloading with 20 mN/min rate.

The NHD was controlled at maximal indentation depth, which was approximately ~1500 nm. Ten NHD readings were recorded for upper surface of each DRC sample in 1-mm-increment from sample’s center, these data being averaged to obtain single NHD. The elastic (Young’s) modulus *E* was calculated for the same load-displacement measuring cycle based on the slope of the upper portion of unloading curve (for the known Poisson’s ratio and elastic modulus of indenter [6, 23]). We understand that absolute values of NHD and elastic modulus *E* determined under such measuring protocol don’t carry strong physical meaning to be reliably compared with DRC probes tested under other measuring protocols [4, 6–11, 18, 19]. But such arranged nanoindentation measurements seem very useful to detect the general appearance of the polymerization kinetics, which can be finalized in most realistic NHD and *E* dependence on light curing time *t*.

The PAL spectra of the polymerized DRC Dipol® samples were registered with a fast-fast coincidence system of 230 ps resolution based on two Photonis XP2020/Q photomultiplier tubes coupled to BaF_2_ scintillator 25.4A10/2 M-Q-BaF-X-N detectors (Scionix, Bunnik, Holland) and ORTEC® (Oak Ridge, TN, USA) electronics [20]. The reliable PAL measurements were performed at highly stabilized conditions (22 °C and relative humidity of 35%) and normal measurement statistics covering 1 million coincidences. The radioactive ^22^Na isotope of low ~50 kBq activity prepared from aqueous solution of ^22^NaCl wrapped by Kapton® foil (DuPont™, Circleville, OH, USA) of 12 μm thickness and sealed) was used as source of positrons sandwiched between two identical DRC samples. The PAL spectra were processed with LT 9.0 program [24], stabilizing an average positron lifetime *τ*_*av*_ as a center of mass of full lifetime spectrum. The accuracies in lifetimes *τ*_*i*_ and intensities *I*_*i*_ were respectively not worse ±0.005 ns and 0.5%.

The best fitting of the measured raw PAL spectra for highly inhomogeneous substances such as polymers or composites is known to be achieved via mixed channels of trapping from defect-specific positron and bound positron-electron states (positronium Ps atoms) [12–17]. This can be resolved due to multi-component fitting with 3 or 4 negative exponentials under free or constrained decomposition (fixed fitting parameters, such as short lifetime maintained close to 0.125 ns [25–27]) and fully normalized intensities Σ*I*_*i*_ = 1.

Because of repulsive interaction with atomic nuclei of environment, positron samples intrinsic regions of minimal charge density, mainly negative or neutral free-volume voids. Describing *positron trapping* in terms of two-state model with only one kind of such defects, which are described by defect-specific intermediate short-lived lifetime *τ*_*2*_ = *τ*_*d*_, the defect-free bulk lifetime *τ*_*b*_, trapping rate in defects *κ*_*d*_, and fraction of trapped positrons *η* can be respectively calculated [12, 13].

Other channel is caused by *Ps decaying*, i.e., positrons annihilating from Ps state as free particles or interacting with electron from environment [12, 13]. In the ground state, the Ps exists as para-Ps (p-Ps, antiparallel spins) decaying intrinsically with two γ-quanta and character lifetime in a vacuum of 0.125 ns, and ortho-Ps (o-Ps, parallel spins) decaying with three γ-quanta and lifetime of 142 ns, these states being occupied with 1:3 relative formation rate. In a matter, since the positron wave function overlapping with electron outside, the annihilation with such electron having an antiparallel spin decreases lifetime to 0.5–10 ns resulting in two γ-rays (“pick-off” annihilation). The Ps localized in free-volume spaces gives indication on their mean radii *R* in terms of longest-lived *τ*_*3*_ lifetime (the intensity of this component *I*_*3*_ correlates well with density of Ps sites):

1 $$ {\tau}_3=0.5\;{\left[1-\frac{R}{R+\varDelta R}+\frac{1}{2\pi}\cdot \sin \left(\frac{2\pi R}{R+\varDelta R}\right)\right]}^{-1}, $$

where Δ*R* = 0.166 nm is fitted empirical layer thickness [13].

By fitting the above equation with measured *τ*_*3*_, the *R*_*3*_, and corresponding free volumes *V*_*f*_ in spherical approximation can be determined. The fractional free volume *F*_*v*_ can be calculated as

2 $$ {F}_v= C\cdot {I}_3\cdot {V}_f, $$

using empirical constant *C* = 0.0018 Å^−3^ [13].

In general, by applying unconstrained x3-term fitting, we cannot correctly parameterize overall positron annihilation process embracing channels originated from both positron- and Ps-trapping, provided essential input from the third (longest-lived lifetime) component [14–16]. Any idealization concerning strict numerical determination of trapping parameters under such conditions remains merely overestimate. Nevertheless, this approach can be well validated for case of kinetics study under condition of the same time-induced influence in all tested parameters.

The protopolymerization kinetics in the studied DRC is assumed to follow single-exponential rule as it can be reasonably expected from previous light-curing research for similar DRC of different thicknesses (tuning in such a way the penetration depth of curing light) [2, 28]. Therefore, this kinetics can be simply presented in the form as relative changes in the control parameter defined by negative exponential serving as normalized relaxation function [29, 30]. Thus, within modeling procedure concerning many experimental points detected, we can characterize the governing kinetics by time constant *τ*, as well as mean square deviations (*err*) of these experimental variables from the normalized modeling curve.

## Results and Discussion

### General Appearance of Photopolymerization Kinetics

**Fig. 2:**
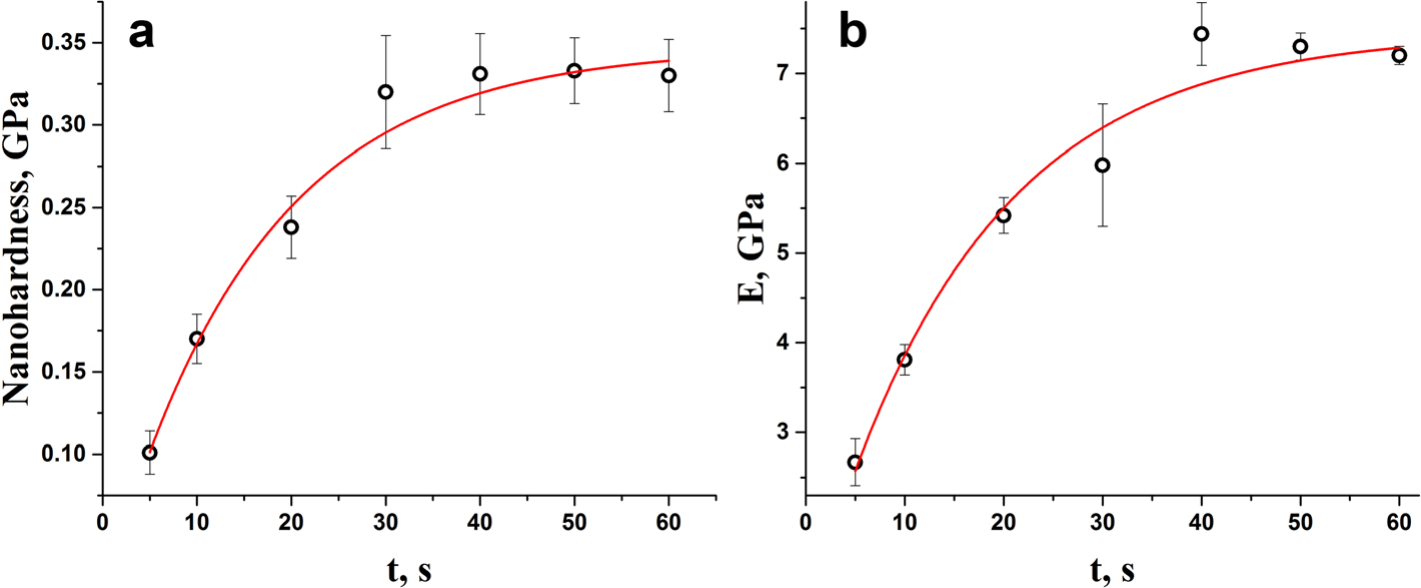
Photopolymerization kinetics in DRC Dipol® via nanohardness (**a**) and elastic modulus *E* (**b**) determined by nanoindentation testing

The photopolymerization kinetics of the DRC Dipol® derived from NHD and elastic modulus *E* measurements is respectively shown in Fig. 2a and b, the fitting parameters describing this kinetics are given in Table 1.

**Table 1:** Fitting statistics describing normalized single-exponential photopolymerization kinetics in different parameters of DRC Dipol®

Control parameter	NHD	*E*	*τ*_*av*_	*τ*_*3*_	*I*_*3*_	*F*_*v*_	*τ*_*2*_	*κ*_*d*_	*η*
Time constant *τ*, *s*	15.0	18.7	17.9	9.1	6.7	18.6	12.1	28.0	27.0
*err.,* ⋅ 10^−3^	1.8	2.8	7.2	1.4	2.9	0.04	15.0	4.1	6.8

In full agreement with previous research [2, 28], the studied kinetics clearly obeys single-exponential functional with best-fitted time constant *τ* approaching 15.0 s for NHD (under saturation level of 0.33 GPa and narrow scattering of experimental variables enveloped within *err* = 1.8⋅10^−3^) and 18.7 s for elastic modulus *E* (under 7.2 GPa saturation and *err* = 2.8⋅10^−3^). This light-curing regime, which can be termed as “weak and long illumination” [28], provides optimal 40 s exposure ensuring deep light-curing as recommended by Dipol® manufacturer [22]. Effect of crosslinking gradient due to light absorbance is estimated to be no more than 80% in difference for top-surface and deep-interior part of the sample, needed to consider this DRC as adequately activated resin [4, 31]. Thus, in harmony with [31], we claim that post-irradiation NHD and *E* increase in the DRC Dipol® is quite commensurable with realistic light-curing kinetics.

### Atomic-Deficient Response in Photopolymerization Kinetics

**Fig. 3:**
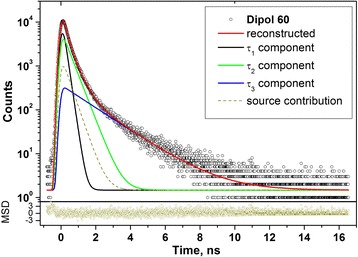
Typical raw PAL spectrum of fully-polymerized DRC Dipol® (exemplified by D60 sample) reconstructed from unconstrained x3-term fitting at the general background of source contribution (*bottom inset* shows statistical scatter of variance)

The PAL spectrum for fully-polymerized D60 sample is depicted on Fig. 3, the similar spectra being detected for other DRC Dipol® samples under research (D5, D10, D20, D30, D40, D50, D60). The raw PAL spectra were parameterized by applying unconstrained x3-term fitting procedure, results for three control samples (D5, D20, D60) being gathered in Table 2.

**Table 2:** The best-fit PAL spectra parameters and trapping modes for DRC Dipol® polymerized under control time intervals within unconstrained x3-term fitting

DRC	PAL spectra fitting parameters						Positron trap-modes			Ps-trap-modes	
	*τ*_*1*_, ns	*τ*_*2*_, ns	*τ*_*3*_, ns	*I*_*2*_, a.u.	*I*_*3*_, a.u.	*τ*_*av*_, ns	*τ*_*b*_, ns	*κ*_*d*_, ns^-1^	*η*, a.u.	*R*_*3*_, nm	*F*_*v*_ *,* %
D5	0.171	0.434	1.851	0.52	0.091	0.459	0.262	2.03	0.345	0.270	1.39
D20	0.165	0.421	1.668	0.53	0.101	0.452	0.257	2.16	0.356	0.255	1.26
D60	0.155	0.411	1.625	0.56	0.101	0.444	0.251	2.45	0.374	0.248	1.17

**Fig. 4:**
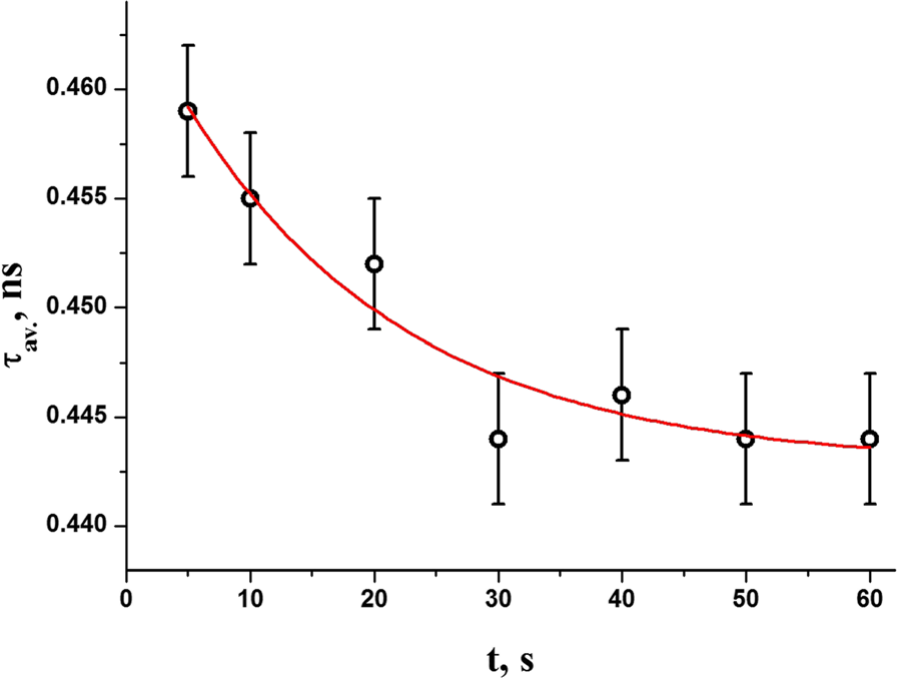
Photopolymerization kinetics in DRC Dipol® determined from average positron lifetime measurements

**Fig. 5:**
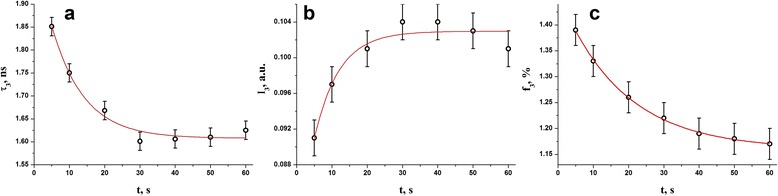
Photopolymerization kinetics in DRC Dipol® determined from o-Ps decaying modes: longest-lived positron lifetime (**a**), third component intensity (**b**), and fractional free volume (**c**)

**Fig. 6:**
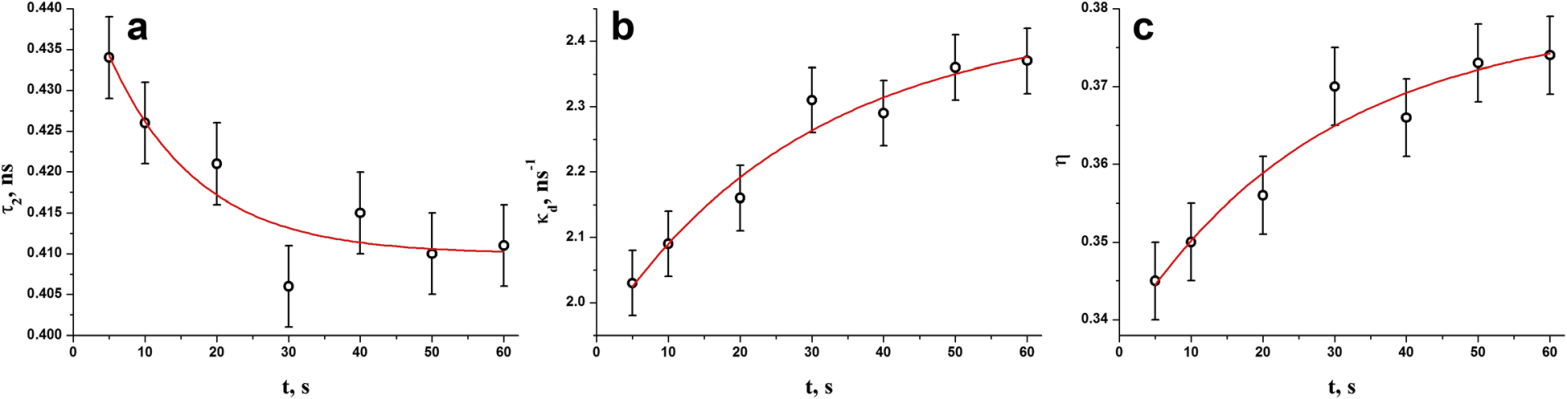
Photopolymerization kinetics in DRC Dipol® determined from positron trapping modes: defect-specific lifetime *τ*_*2*_ (**a**), trapping rate in defects *κ*_*d*_ (**b**), and fraction of trapped positrons *η* (**c**)

It should be emphasized that average positron lifetime *τ*_*av*_ decreases during polymerization, along with both longest-lived *τ*_*3*_ and defect-specific *τ*_*2*_ lifetimes, while *I*_*3*_ and *I*_*2*_ intensities reveal an opposite growing tendency. More generally, the volumes of holes responsible for positron and Ps trapping are reduced during light curing, while their content obeys an obvious growing tendency. These features result in near-monotonic dependencies for other PAL parameters, e.g., increasing trend in positron trapping rate in defects *κ*_*d*_ and fraction of trapped positrons *η*, decreasing trend in bulk defect-free positron lifetime *τ*_*b*_, free-volume hole radius *R*_*3*_ estimated in respect to Eq. (1) and fractional free volume *F*_*v*_. The photopolymerization kinetics for these parameters are respectively given on Figs. 4, 5, and 6 for average lifetime *τ*_*av*_, as well as both o-Ps and positron trapping modes, the parameters of normalized single-exponential fitting statistics being gathered in Table 1.

In respect to the values of time constants *τ* describing single-exponential light-curing in DRC Dipol® due to growing NHD (*τ* = 15.0 s) or elastic modulus (*τ* = 18.7 s), the most adequate photopolymerization kinetics can be derived from time dependence of average positron lifetime *τ*_*av.*_ defined as mass center of whole PAL spectrum (*τ* = 17.9 s), and fractional free volume *F*_*v*_ (*τ* = 18.6 s) calculated with Eq. (2). Both these parameters seem meaningful to describe realistic kinetics.

It is known that cross-linking in dimerthacrylate-based monomer systems predominated by chain reaction polymerization is the main consequence resulting from light-curing [4, 5, 8, 32]. Many of physical properties of DRC are essentially influenced by conversion of carbon-carbon double bonds in these monomers into extended single-bond network of light-cured polymer composite. At a microscopic level, the degree of conversion in monomer-polymer system results in increased NHD and elastic modulus *E*, which can be accepted (under applied measuring conditions) as principal determinant of the generalized photopolymerization kinetics. These processes are accompanied by some changes in the DRC free-volume structure, thus being reflected in the PAL spectra.

Undoubtedly, the channel of Ps-trapping determined by fractional free-volume *F*_*v*_ plays a decisive role in this kinetics in view of narrow scattering of experimental variances *err* = 0.04⋅10^−3^ (Table 1). These changes occur under light exposure mainly due to polymer chain cross-linking in DRC, causing appearance of ever smaller voids owing to fragmentation of Ps-trapping sites [32–35]. Noteworthy, the sizes of Ps-trapping free-volume holes *R*_*3*_ (~0.24–0.27 nm) defined by longest-lived lifetime *τ*_*3*_ due to Eq. (1) and their content expressed in *I*_*3*_ intensity show more than double-time quicker kinetics (as it follows from time constants in Table 1). It means that photopolymerization kinetics in the studied DRC is not defined by Ps-trapping solely, other competitive processes especially at higher photoexposures taking place. We believe such additional process is positron trapping occurring in the photopolymerized DRC, which demonstrates more than double-time stretched kinetics for both trapping rate in defects *κ*_*d*_ and fraction of trapped positrons *η* (Table 1). Near filler particles, the fragmented o-Ps traps convert in interfacial voids (i.e., triple junctions) representing themselves as free-volume pseudogap holes at the interface between the outer surface layer of filler particles and innermost layer of polymer [14, 15, 36]. These free-volume voids are also efficient trapping sites for positrons corresponding to increased fraction *η* in light-cured DRC.

Thereby, the fragmentation of Ps-traps accompanied by partial Ps-to-positron traps conversion governs photopolymerization kinetics in the studied DRC. That is why the light-curing kinetics described by changes in average positron lifetime *τ*_*av.*_, which serves as most adequate determinant of generalized PAL spectra (despite applied PAL-spectra-fitting procedures) [12, 13], obeys an effective time constant *τ* very close to nanoindentation-controlled polymerization kinetics (changes in NHD and *E*, see Table 1). Correspondingly, appearance of a few single-exponential relaxation processes with somewhat different time constants enhances the scattering of experimental variables resulting in higher *err* = 7.2⋅10^−3^ in the light-curing kinetics.

## Conclusions

This research is aimed to justify photopolymerization light-curing kinetics in dimethacrylate-based polymer/filler dental composites Dipol® revealed in its general macroscopic appearance through nanoindentation study and intrinsic nanoscale volumetric characteristics due to atomic/subatomic free-volume voids obtained using lifetime spectroscopy of annihilating positrons.

Nanoindentation measurements testify that polymerization in the studied dental composites is saturated after 30–40 s of illumination from LED source (emitted light in 420–480 nm range with ~900 mW/cm^2^ output power density), showing single-exponential kinetics with character time constant close to 15.0 and 18.7 s for nanohardness and elastic modulus, respectively. This photopolymerization kinetics was examined through atomic-deficient characteristics extracted from positron lifetime spectra parameterized employing unconstrained x3-term fitting procedure. The most adequate kinetics response was found in light-curing process determined for mass center of whole positron lifetime spectrum (i.e., average positron lifetime) and fractional free volume of Ps-trapping sites. This correlation proves that fragmentation of free-volume Ps-traps accompanied by partial Ps-to-positron traps conversion determines the light-curing volumetric shrinkage in dental resin composites Dipol®.

## Abbreviations

BisGMA: Bisphenol A-diglycidyl dimethacrylate
D3MA: Decanediol dimethacrylate
DRC: Dental restorative composite
NHD: Nanohardness
PAL: Positron annihilation lifetime
TEGDMA: Triethyleneglycol dimethacrylate
UDMA: 1,6-bis[2-(methacryloyloxy)ethoxycarbonylamino]-2,4,4-trimethylhexane
